# Reconstructions of individual fish trophic geographies using isotopic analysis of eye-lens amino acids

**DOI:** 10.1371/journal.pone.0282669

**Published:** 2023-03-16

**Authors:** Amy A. Wallace, Greg S. Ellis, Ernst B. Peebles

**Affiliations:** 1 College of Marine Science, University of South Florida, St. Petersburg, Florida, United States of America; 2 Johns Hopkins All Children’s Hospital, St. Petersburg, Florida, United States of America; MARE – Marine and Environmental Sciences Centre, PORTUGAL

## Abstract

Fish eye lenses are a proteinaceous structure that grows by accumulating layers in a chronological manner. Each layer becomes metabolically inert, capturing the ratio of heavy/light carbon and nitrogen isotopes at time of formation. Therefore, eye lenses contain chronological isotopic records and can be used to create a temporal isotopic history throughout an individual’s lifetime. We analyzed eye lens amino-acid δ^15^N to address spatio-temporal baseline variability and to reconstruct trophic histories of 10 individual Red Snapper. Proteins from sequential eye lens laminae were derivatized to measure 10 amino acids, from which glutamic acid (trophic) and phenylalanine (source) were used to estimate trophic positions at different points in life. Best-fitting regressions were generated to represent individual (R^2^ ≥ 0.89) and generalized (R^2^ = 0.77) trophic trajectory for Red Snapper. The resulting trophic trajectories indicated an increase in trophic position with increasing length. Until recently, there has not been a lifetime isotopic structure with enough organic nitrogen to recreate geographic histories using compound-specific stable isotope analysis of amino acids (CSIA-AA). This study confirms that eye-lens laminae can be used to reconstruct trophogeographic histories via CSIA-AA.

## Introduction

The bulk stable isotope analysis (SIA) approach to ecosystem studies has resolved some of the challenges prevalent in stomach-content and feeding-observation studies, yet bulk SIA presents its own complications and challenges. One challenge is spatio-temporal variability in baseline nitrogen isotopes and another is that trophic fractionation variability is not always consistent with previously published values [1,2]. Compound-specific isotope analyses of amino acids (CSIA-AA) is a method that requires fewer assumptions than bulk SIA, and thus it has become a powerful analytical tool for ecological interpretations [2,3]. Based on patterns of nitrogen isotope fractionation, AAs can be categorized as “trophic”, “source”, or “others” [4]. Trophic AAs fractionate with increasing trophic position as a result of their frequent involvement in deamination and transamination reactions. In contrast, source AAs are minimally fractionated as trophic level increases [2,5]. Other AAs do not fractionate consistently with trophic position and so yield little useful information unless further analytical investigations are employed [4]. Analysis and comparison of trophic and source AAs has improved both the precision and accuracy of trophic position estimates (TP_CSIA_) [2,4,6,7]. Currently, CSIA-AA is commonly used on soft tissues of specimens, which gives an isotopic signature at the time of collection that is based on tissue turnover rate (the time it takes for a tissue to incorporate isotopic changes). However, using CSIA-AA on fish eye lenses can potentially create a temporal history of amino-acid isotopes throughout most of an individual organism’s lifetime.

Fish eye-lenses consist of metabolically-inert, protein-rich layers (laminae) of lens fiber cells. Lens fiber cells are a product of the lens epithelium, a single layer of living cells that surrounds most of the eye lens [8,9]. During post-embryonic lens growth, lens epithelium cells at the lens equator transform into lens fiber cells by filling with crystallin (a structural protein), elongating towards the lens poles, and then removing their organelles (and any associated DNA) through attenuated apoptosis. During typical apoptosis, the cell dies and the entire cell disintegrates. However, attenuated apoptosis differs from typical apoptosis in the sense that the cell membrane and internal proteins remain. Attenuated apoptosis effectively stops further protein synthesis by disintegrating organelles and DNA within the new lens fiber cells, rendering the cells metabolically inert in a manner analogous to hair, feathers, and hooves [8,9]. Prior to attenuated apoptosis, while the cells are elongating until ultimately meeting at the poles of the eye lens, a layer, or lamina, is formed that contains multiple layers of lens fiber cells. Each new lamina is formed in the diametrical periphery of the previous ones, creating concentric layers, with the oldest material in the center of the lens. Because non-embryonic eye lenses can form within a few days after fertilization [9], the isotopes within the lens laminae capture information that dates throughout the post-embryonic life of the individual fish [10,11]. Unlike otolith increments, each lamina does not have a defined temporal value associated with it. However, delamination techniques allow isotopic reconstructions at a frequency of approximately two to three months [12]. It is also worth noting that because the lens grows as the fish grows, there is a positive correlation between lens diameter and fish length, and this relationship is often isometric [15–17].

The present paper addresses spatio-temporal variability in baseline nitrogen using a larger mesopredator, Red Snapper (*Lutjanus campechanus*). Previous work by Harada et al. [13] used stable-isotope analysis of eye-lens amino acids to calculate lifetime trophic positions of chub mackerel, a dedicated zooplanktivore, to suggest an increase in trophic position occurred during early life (i.e., its trophic trajectory was positive). We investigate whether CSIA-AA of eye lens laminae can be used to track trophic position changes and geographic movement over an individual fish life-history, which is known to change trophic position during life [3–5]. Individual trophic trajectories were created for each fish and compared to published trends. We also discuss the utility of CSIA-AA of eye lens laminae for reconstructing geographic histories.

## Methods and materials

### Field work/sample collection

Two sample sites, 8–40 and 4–40, were selected because they are geographically separated along a well-documented nitrogen isotope gradient on the West Florida Shelf [11,14]. Site 8–40 is located near the Alabama/Florida state lines on the northern edge of the West Florida shelf. Site 4–40 is further south on the West Florida Shelf, off-shore of Tampa Bay. As individual fish move along this isotope gradient, changing baseline δ^15^N should be reflected as differences in baseline nitrogen present in chronological eye lens isotope records. Fish samples were collected during August 2014 from the R/V *Weatherbird II* using longline fishing techniques. At each station, 8 km of 544 kg-test monofilament was deployed from the ship as the mainline, with approximately 500 baited hooks attached to the mainline via gangions. Gangions were 3.7 m long and used 91 kg test line and #13 circle hooks that were baited alternately with cut fish (Atlantic Mackerel, *Scomber scombrus*) and various species of cut squid. Fish were euthanized by decapitation or cervical dislocation followed by pithing.

Whole Red Snapper eyes were removed using a knife in the field and were kept frozen between -20 and -40°C until the time of lens dissection (delamination). Lenses were delaminated using forceps under a dissecting microscope according to a modification of the technique described in Wallace *et al*. [10]. In this modification, a water rinse was not used to remove the lens capsule; instead, it was cut away using forceps and a scalpel. This change to the processing method ensured there was no dissolution of the outermost lamina, which is highly water soluble. Before and after each lamina removal, lens diameter was measured to the nearest 0.05 mm using an ocular micrometer. Diameters were converted to radii, and the laminar radial midpoint (LRM, in mm) was calculated as the average of the two radius measurements (before and after) taken during eye lens delamination per Wallace *et al*. [10].

### AA-CSIA processing

Amino acids (AA) are difficult to separate using gas chromatography because they are insufficiently volatile and have a large number of functional groups. Therefore, AAs require derivatization prior to isotopic analysis [15]. All samples were derivatized using the methods outlined in Ellis 2012 [16–18], which is described here in brief.

For each individual lamina, a dry weight of approximately 1 mg was placed in a 20 mL scintillation vial with 2 mL 6 M HCl and heated at 100°C for 24 hours to hydrolyze proteins. The acid solution was evaporated under a stream of N_2_ at 70°C, after which samples were re-suspended in 0.05 N HCl. The digested sample was then transferred to a Dowex 50wx8, 200–400 mesh cation-exchange resin column assembled in a clean Pasteur pipette. Non-AA components were flushed from the column using deionized water. The AAs were then eluted from the Dowex resin column using 3 M NH_4_OH. Next, the eluent was evaporated to dryness in a drying oven at 70°C. The dry AA samples were then esterified using 2 mL of anhydrous isopropanol acidified with acetyl chloride (4:1) at 100°C for one hour. After this step, the esterified AAs were evaporated to dryness under a N_2_ stream, followed by acylation by adding a solution of acetone, trimethylamine, and acetic anhydride (5:2:1 by volume) and heating at 60°C for 10 minutes. The acylated AA samples were again evaporated to dryness under an N_2_ stream. The acylated AA samples were then re-dissolved using 2 mL ethyl acetate. Approximately 1 mL of NaCl-saturated water was added to the solution and the organic phase was extracted and evaporated to dryness under an N_2_ stream. All samples were refrigerated until injection into the gas-chromatography combustion isotope-ratio-mass-spectrometer (GC-C-IRMS). Prior to injection into the GC-C-IRMS, the derivatized samples were re-dissolved in 1 mL ethyl acetate and 50 μL was transferred to a low-volume glass autosampler vial insert.

CSIA-AA is relatively expensive and time consuming. Because Red Snapper have periods of high site fidelity, only six laminae per fish (10 fish) were selected for CSIA-AA. Each lens yielded between 14 and 23 laminae. All core and second laminae were analyzed to capture data corresponding with an ontogenetic shift during early life. The other four laminae analyzed were equally spaced throughout the remaining laminae. Each sample’s (n = 60) ^15^N/^14^N ratio was measured in replicate using an Agilent 6890 GC and Thermo Finnigan GCC-III interface coupled with a continuous-flow Thermo Finnigan Delta+XL isotope ratio mass spectrometer at the University of South Florida College of Marine Science in St. Petersburg, Florida. For CSIA-AA, all results are presented in standard notation (δ, in ‰) relative to air

$$\delta^{15}N=\left(\frac{R_{sample}}{R_{standard}}-1\right)\;x\;1000,$$

where R is the ratio ^15^N/^14^N. Replicate isotope measurements were averaged prior to data analysis.

### Trophic positions

A trophic position for each lamina was derived using the following equation:

$$TP=\frac{{(\delta }^{15}N_{trophic}-\delta^{15}N_{source})-\beta }{TDF}+1,$$

where δ^15^N_trophic_ and δ^15^N_source_ are the δ^15^N of the representative trophic and source AAs, TDF is the calculated trophic discrimination factor, and β is the Δ δ^15^N between the source and trophic AAs at the primary-producer level [6]. We used glutamic acid and phenylalanine as our representative trophic and source AAs, respectively. For this study, we used TDF = 5.7‰ and β = 3.6‰ according to Bradley *et al*. [6].

After calculating trophic position for the six eye-lens laminae, trophic position data for each laminar radial midpoint were interpolated using a best-fitting regression model, producing trophic trajectories. Trophic trajectory is defined here as changes in trophic position during the life of the individual, a concept that has also been referred to as trophic growth [6,10]. The best-fitting regressions for each fish was determined using the Comparison of Alternative Models routine in Statgraphics Centurion (v. 18, Statpoint Technologies, Inc., Warrenton, VA).

### Fish length estimation at lens radial midpoint

Eye lenses grow continuously during life of the fish and have a strong positive correlation with the length of the fish [19–21]. Fish standard length (SL) at specific LRMs (SL_LRM_) was calculated using the equation

$${SL}_{LRM}={SL}_{Max}(\frac{LRM}{{LRM}_{MaxRad}}),$$

where SL_Max_ is the standard length at capture and Lens_MaxRad_ is the radius of the intact lens before delamination.

### Otolith aging

These Red Snapper were aged as part of a previous growth study [22]. The methods for determining ages are outlined in Herdter *et al*. [22].

### Data location and ethics statement

All fish collections and tissue dissections were supported by research collecting permits and Institutional Animal Care and Use Committee (IACUC) protocols at the University of South Florida (IACUC protocol IS00000515).

## Results

### CSIA-AA of eye lens amino acids

**Fig 1:**
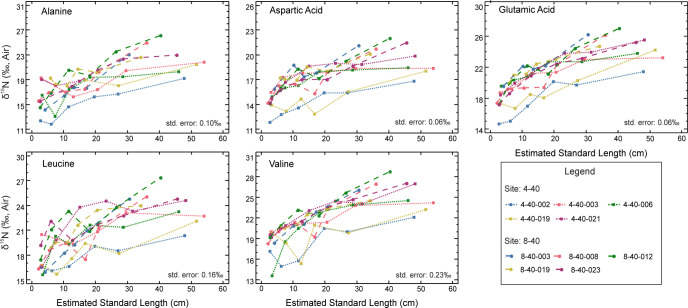
Trophic AAs at estimated fish lengths. Each line represents an individual. Squares are Red Snapper from SL 4–40 and circles are Red Snapper from SL 8–40. Standard error is from Table 1. Not all AAs have a standard error because not all AA present in eye lens protein were able to be measured in muscle protein.

**Fig 2:**
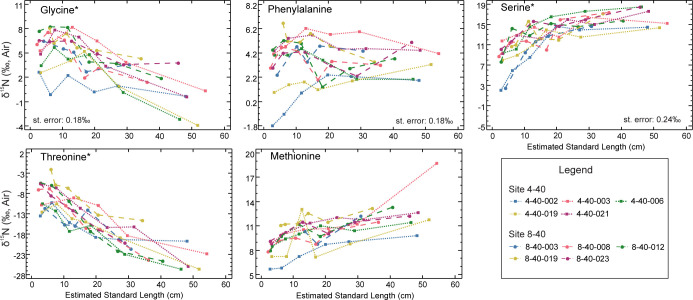
Source and other (indicated by *) AAs at estimated fish lengths. Each line represents an individual. Squares are Red Snapper from 4–40 and circles are Red Snapper from 8–40. Standard error is from Table 1. Not all AAs have a standard error because not all AA present in eye lens protein were able to be measured in muscle protein.

The Red Snapper eye lenses in this study ranged between 9.7 mm and 11.0 mm in diameter. Each lens yielded between 14 and 23 laminae for analysis, but only six were chosen for each fish because the Red Snapper is considered to have a moderate degree of site fidelity (Table 1). Isotopic values were obtained for 13 AAs, but only 10 are reported here (five trophic AAs, three source AAs, and two “other” AAs; see Figs 1 and 2). Isoleucine and proline are not reported because these AAs care subject to interferences from close-eluting peaks of leucine and serine, respectively. Lysine is also not reported because it was not consistently resolved during analyses for these samples. In Figs 1 and 2, isotope values are plotted against estimated standard length. All of the AAs measured had considerable variability among laminae within individual eye lenses. The reported standard error for each AA was based on CSIA-AA of King Mackerel (*Scomberomorus cavalla*) muscle from eight successive runs; these are presented in Table 2. The data is Table 2 demonstrate the reproducibility of our methods and are not intended for use as a comparison of muscle and eye lens protein AA composition. Because they are two different tissues, it is assumed the AA composition will be different among these tissues.

**Table 1:** Individual Red Snappers used in the CSIA-AA.

Fish ID	Age (years)	Standard Length (cm)	Lens Radius (mm)	Laminae Dissected
4-40-002	5.9	55	11.0	18
4-40-003	6.6	59	10.9	25
4-40-006	5.7	54	10.5	15
4-40-019	6.6	59	11.0	16
4-40-021	6.1	56	10.8	17
8-40-003	3.5	38	9.7	14
8-40-008	3.8	41	9.8	14
8-40-012	4.6	47	10.3	17
8-40-019	4.0	42	10.4	15
8-40-023	5.2	51	10.5	21

**Table 2:** Isotopic measurement error for nine of the 13 AAs used in the present study.

Replicate ID	Alanine (‰)	Glycine (‰)	Valine (‰)	Leucine (‰)	Serine (‰)	Aspartic Acid (‰)	Glutamic Acid (‰)	Phenylalanine (‰)	Lysine (‰)	Caffeine (‰)
Acq 62	24.0	-6.1	22.5	21.4	-4.9	23.8	23.2	0.9	-1.2	-3.7
Acq 63	24.3	-4.8	23.2	22.9	-3.9	23.7	23.2	1.1	1.1	-3.7
Acq 64	23.4	-5.2	24.0	22.0	-2.6	24.1	23.3	1.2	1.2	-3.9
Acq 65	24.0	-5.5	22.8	22.5	-2.6	24.1	23.3	1.2	1.2	-3.9
Acq 66	24.2	-5.8	24.1	22.1	-4.1	23.9	23.4	2.1	1.5	-3.6
Acq 67	23.9	-4.8	23.5	22.2	-4.3	23.7	23.1	1.2	1.3	-4.3
Acq 68	24.4	-4.8	22.6	22.1	-4.4	23.9	23.1	0.7	0.6	-3.9
Acq 69	23.9	-4.9	22.6	21.8	-4.5	23.8	23.0	0.3	1.9	-4.0
*Mean*	*24*.*0*	*-5*.*2*	*23*.*2*	*22*.*1*	*-4*.*1*	*23*.*9*	*23*.*2*	*1*.*1*	*0*.*8*	*-3*.*9*
*Standard Deviation*	*0*.*3*	*0*.*5*	*0*.*6*	*0*.*5*	*0*.*7*	*0*.*2*	*0*.*2*	*0*.*5*	*1*.*0*	*0*.*2*
*Standard Error*	*0*.*1*	*0*.*2*	*0*.*2*	*0*.*2*	*0*.*2*	*0*.*1*	*0*.*1*	*0*.*2*	*0*.*4*	*0*.*1*

δ^15^N in each AA was measured from eight muscle samples from a single King Mackerel. Muscle protein has a different AA composition than eye-lens crystallin protein and therefore there is a difference in AAs resolved in GC-C-IRMS runs presented in this table when compared to AA presented in Table 3. The purpose of this table is to indicate the reproducibility of the derivatization process and not to directly compare the AA compositions of eye lens protein and muscle protein. Results for a laboratory working standard of caffeine (a compound free of possible derivatization effects) are also presented.

The ranges of AA δ^15^N are given in Tables 3 and 4. All "trophic” AAs generally increased with increasing fish body length. Threonine, which is classified as an “other” type of AA due to isotopic patterns, had the largest range, varying from -26.7 to -2.1‰. Threonine decreased across all laminae as length increased, which is an opposite δ^15^N pattern when compared to that of “trophic” AAs and is consistent with previous reports [2,4]. The smallest range was observed for phenylalanine (a “source” AA), which varied from -1.8 to 6.7‰. When comparing the two sample sites, 4–40, which is located off of the middle West Florida Shelf, exhibited δ^15^N maximum and minimum values for each AA that were generally lower than 8–40 which is located on the north West Florida Shelf.

**Table 3:** Amino acid δ^15^N values (δ, in ‰) for Red Snapper individuals from station 4–40.

Sample ID	Ala (T)	Asp (T)	Glu (T)	Leu (T)	Val (T)	Met (S)	Phe (S)	Gly (O)	Ser (O)	Thr (O)
4–40 (all fish)	11.8–21.8	11.8–19.9	14.7–25.5	15.6–24.6	13.5–27.0	5.7–18.7	-1.8–6.22	-3.9–8.2	2.0–19.9	-16.7 - -5.7
4-40-002	11.8–19.2	11.8–16.8	14.7–21.5	16.1–20.3	14.9–22.1	5.7–9.8	-1.8–2.4	-0.4–2.6	2.0–14.7	-19.8 - -10.3
4-40-003	16.2–21.8	15.5–18.4	18.7–23.2	19.3–23.1	20.0–24.2	8.9–18.7	4.1–6.2	0.3–8.2	11.7–16.4	-22.8 - -9.1
4-40-006	13.1–20.3	15.0–18.4	19.6–23.8	15.6–23.2	13.5–24.5	8.6–11.4	2.0–4.6	-3.2–5.7	9.4–18.5	-26.7 - -10.6
4-40-019	18.1–21.5	12.8–18.1	16.7–24.2	15.7–22.1	15.4–23.2	7.2–13.1	0.9–3.3	-3.9–5.2	8.0–14.4	-26.6 - -10.6
4-40-021	18.0–19.0	14.8–19.9	18.4–25.5	16.6–24.6	19.2–27.0	8.8–12.7	4.4–5.7	-0.4–7.8	9.3–17.6	-26.0 - -5.7

Site 4–40 is the more southern site in this study. Each amino acid represented by the minimum and maximum, where Ala = alanine, Asp = aspartic acid, Glu = glutamic acid, Leu = leucine, Val = valine, Met = methionine, Phe = phenylalanine, Gly = glycine, Ser = serine, and Thr = threonine.

**Table 4:** Amino acid δ^15^N values (δ, in ‰) for Red Snapper individuals from station 8–40.

Sample ID	Ala (T)	Asp (T)	Glu (T)	Leu (T)	Val (T)	Met (S)	Phe (S)	Gly (O)	Ser (O)	Thr (O)
8–40 (all fish)	14.2–26.1	14.1–22.0	17.2–27.1	16.0–27.4	18.3–28.7	7.9–13.3	1.4–6.7	1.4–8.2	12.5–21.2	-24.6 - -2.1
8-40-003	14.2–23.0	15.9–21.2	19.5–26.2	16.0–24.8	18.4–26.1	8.8–12.2	2.4–4.8	2.7–6.5	2.5–14.6	-21.6 - -11.6
8-40-008	15.6–25.0	14.2–20.7	17.4–26.2	16.4–25.0	18.3–27.0	7.9–11.5	2.0–4.7	1.4–7.6	8.7–17.4	-24.3 - -6.8
8-40-012	14.5–26.1	14.1–22.0	17.2–27.1	17.4–27.4	19.2–28.7	8.0–13.3	1.4–5.2	1.9–8.2	7.7–15.7	-24.6 - -5.6
8-40-019	18.0–22.6	17.0–20.2	20.0–24.8	18.9–24.0	20.5–24.5	11.1–13.2	3.5–6.7	4.3–8.1	10.7–17.1	-14.7 - -2.1
8-40-023	15.5–23.0	14.2–21.5	17.2–25.3	19.2–24.8	19.6–27.0	9.1–12.2	2.3–5.1	3.6–6.6	10.1–18.4	-20.2 - -5.6

Site 8–40 is the more northern site in this study. Each amino acid represented by the minimum and maximum where Ala = alanine, Asp = aspartic acid, Glu = glutamic acid, Leu = leucine, Val = valine, Met = methionine, Phe = phenylalanine, Gly = glycine, Ser = serine, and Thr = threonine.

### Lifetime trophic position reconstruction

**Fig 3:**
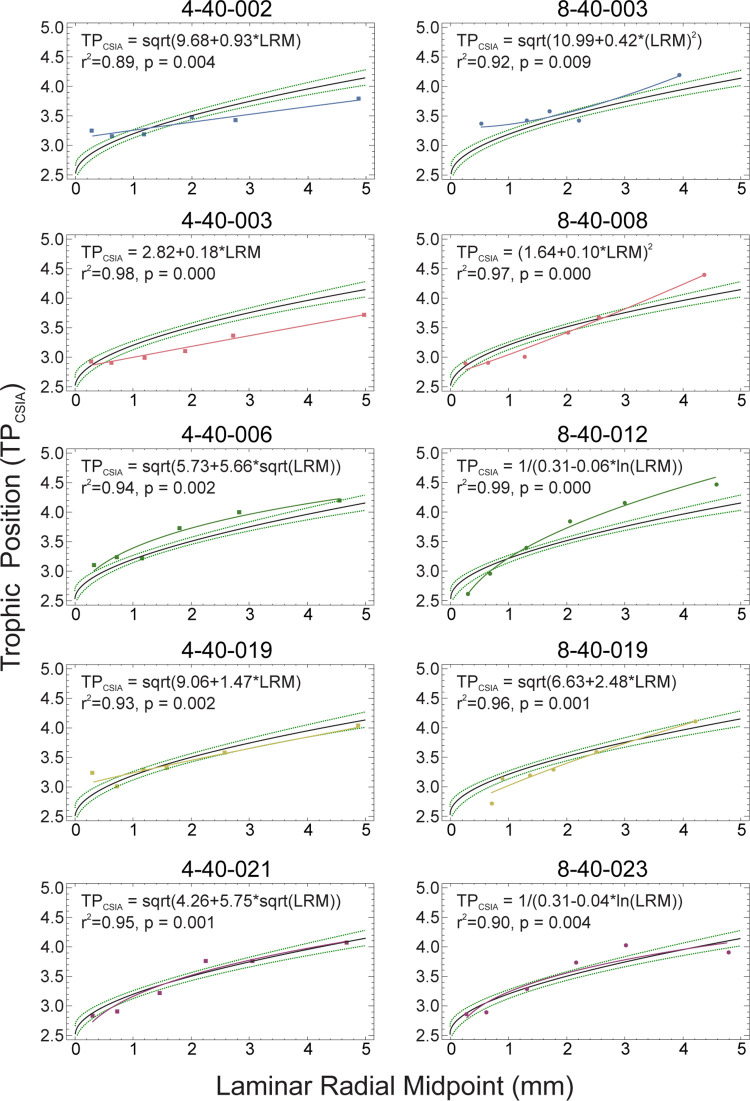
Comparison of composite trophic trajectory model with individual trophic trajectory models. The squares and circles are TP_CSIA_ for individual laminae, colored solid line is the individual model, black solid line is the composite model calculated using all TP_CSIA_ values, and green dotted lines are the 95% confidence intervals for predicted means of the composite model is TP_CSIA_ = (1.587 + 0.201 * sqrt(LRM))^2^, n = 60, R^2^ = 0.77, p < 0.0001.

Individual trophic trajectory regressions (Fig 3) for Red Snapper had R^2^ ≥ 0.89 and the best-fitting model for all Red Snapper individuals, when treated as an aggregate had R^2^ = 0.77. Individual trophic trajectory regressions were used to interpolate trophic position for each laminar radial midpoint not empirically measured; the resulting curves represent the trophic trajectories of individual Red Snapper.

## Discussion

Fish eye lenses were established as likely lifetime isotope recorders by Wallace *et al*. [10], and have since been used to study various teleosts and elasmobranchs [21,23–25]. Unlike the previous stable isotope studies which focus on bulk stable isotope data, we use CSIA-AA to reconstruct trophic life histories for individual fish. Although δ^15^N was measured for 13 AAs, only 10 are presented here. Lysine, isoleucine, and proline were excluded because lysine proved difficult to resolve across most laminae and isoleucine and proline tended to partially coelute with leucine and serine, as the peaks are approximately 10–20 seconds apart chromatographically. Partial peak-overlap can cause isoleucine and proline to be measured before background levels return to baseline values from the previous measurement. This degree of partial overlap causes the subsequent AA to be measured incorrectly, and in most cases this minimally affects the previous AA measurement. During peak integration, the first part of the peak is dominated by isotopes that are heavier relative to those encountered near the end of the peak [26]. Therefore, the heavy isotopes ratios from the second peak can mix with the light isotopes from the previous peak, causing much larger errors in measurement. However, these AAs, and in fact all AAs, can be targeted and better resolved using derivatization and GC-C methods optimized specifically for specific AAs but this may be achieved at the risk of not resolving other AAs [17]. The current study targeted glutamic acid and phenylalanine while chromatographically separating other AAs.

Lifetime isotopic profiles were separated into their respective categories, identified by Whiteman *et al*. [4] as “trophic,” “source,” and “other.” Figs 1 and 2 demonstrate isotopic variability within an individual eye lens as well as among eye lenses from multiple individuals. As expected due to trophic fractionation, the trophic AA δ^15^N values were considerably higher than those of source AAs and had a tendency towards having higher values with increasing fish length and age. However, variability was also present among the source AAs within individual lenses. Considering the study organisms are reef fish with presumably high site fidelity, this was unexpected. The most probable explanation for source AA variability is changing baseline δ^15^N values caused by geographic differences (i.e., baseline) or possibly water-mass movement. Fish can feed on different prey types (some of which may move) according to availability, seasonality, etc., potentially creating shifts in the “apparent baseline” isotopic values of source AAs across an individual’s lifetime [27,28]. While capture-recapture and tagging data may suggest high site fidelity over short period of life (i.e., between mark and recapture), the AA isotopes provide a more complete life-history tracer of trophic geography and corroborate the tagging data that suggests some Red Snapper travel hundreds of kilometers during their lifetimes [29,30].

Of the two locations, 8–40 is located farther north and west in the Gulf of Mexico, near the Florida-Alabama border. All AA δ^15^N ranges (with the exception of leucine) started and ended at higher values for 8–40 fish than those of fish located farther south on the West Florida Shelf at 4–40 (Tables 3 and 4). This is consistent with observed and modeled isoscapes (maps showing spatial trends of isotopic composition) in the Gulf of Mexico [11,14]. Radabaugh *et al*. [14] identified a nitrogen isotopic gradient on the West Florida Shelf with lower δ^15^N values to the south and higher δ^15^N values to the north. This gradient exists because of the processes governing nitrogen sources and cycling into the different areas. The southern part of the WFS is has a greater extent of nitrogen fixation by diazotrophs (such as *Trichodesmium*) and the northern Gulf has larger inputs of terrigenous nitrogen from the Mississippi, Atchafalaya, and Mobile Rivers. The two dominating processes create a latitudinal/longitudinal gradient along the West Florida Shelf that is seasonally and annually robust [14,31,32]. Radabaugh *et al*. [14] used bulk isotope data (fish muscle from multiple species) to create their isoscapes, and the first-order trend in Red Snapper AA isotopes is consistent with their reported trends.

Bulk isotope data has a limitation in that changing baseline δ^15^N can confuse isotopic interpretation while calculating higher trophic levels. This is usually counteracted by conducting bulk δ^15^N analysis on not only the consumer but its possible diet items [33,34]. CSIA-AA eliminates the need to isotopically measure all food-web components and has been used to disentangle the effects of (isoscape) baseline variations that propagate through higher trophic positions [2,34,35]. Instead, eye-lens CSIA-AA directly documents lifetime baseline isotopic variation among individuals, lessening dependence on spatial-stationarity assumptions that are used while making trophic-position estimates [36].

Red Snapper is a commercially and recreationally important reef-fish species in the Gulf of Mexico that has undergone years of research and stock assessments. Between the 1980s and early 2000s, Red Snapper were highly depleted because of overfishing, but the stock has shown signs of rebuilding with no overfishing in recent years [37]. Red Snapper associate with reef habitat from as soon as they leave the planktonic stage (settlement) until at least age eight [38]. During this time, Red Snapper grow rapidly and their habitat complexity generally increases with fish length [30,39,40]. It is well documented that Red Snapper are euryphagous, consuming whatever fish and invertebrates are readily available [38]. In the northeastern Gulf of Mexico, stomach-content analysis has found that prey includes fish, crabs, pelagic zooplankton, and mantis shrimp [41]. The dominant prey foraged by Red Snapper can change by season, depth, or fish length [41].

The Red Snapper is a species of reef fish that is considered to have periods of high site fidelity during its life history [29,42,43]. Examining lifetime profiles in the source AAs (Fig 2), two individuals from the more southerly location (fish 4-40-002 and 4-40-019) appear to have originated from even farther south, where isoscapes reflect an environment dominated by nitrogen-fixing cyanobacteria that occupy oligotrophic waters [11,14,32]. In general, the methionine profiles suggest a number of individuals moved north, occupying areas with elevated baseline δ^15^N later in life, and the phenylalanine profiles suggest a combination of northward, southward, and neutral movement. Further investigation into the difference between these two source AAs and their specific patterns would be beneficial. For fish collected from a single location, it is expected that laminar isotopic values become more similar as newer laminae reflect the common isotopic baseline for that location. Outliers such as the outermost methionine measurement in fish 4-40-003 could represent recent arrival from another area. However, these data are consistent with the idea that movement may be very important to Red Snapper ecology. In a Texas capture/recapture study, Red Snapper that moved had a higher average daily growth rate than those that remained in place, suggesting it is beneficial for Red Snapper to move [44].

Our trophic-position estimates produced a trophic trajectory curve (Fig 3) that appears to be reasonably general in nature, thus indicating a positive correlation between trophic position and length [45]. Upon estimating trophic trajectory curves for individual Red Snapper, it became apparent that individuals may take somewhat different paths toward reaching their respective, maximum trophic positions. Some Red Snapper experienced linear trophic trajectory, whereas others experienced nonlinear (e.g., square-root model) trophic trajectories (Fig 3). The different trophic trajectories for individual Red Snappers support the previously published diet studies that suggest Red Snapper eat a variety of prey items [41]. While the statistical fit of the generalized (composited individuals) trophic trajectory curve is lower than those for individuals (R^2^ = 0.77 vs. R^2^ > 0.88), the composite model is likely a reasonable first-order approximation that could be applied to the trophic trajectory of Red Snapper in other parts of the species’ range.

Bulk isotope data reflect a combination of trophic and geographic influences; if one can remove the trophic influence, then the geographic influence remains. Until recently, there has not been a lifetime isotopic recorder with enough organic nitrogen to recreate geographic histories using CSIA-AA. In this regard, this study confirms that eye-lenses are a useful isotopic archive for CSIA-AA. However, there is one complication to the CSIA re-creation of geographic histories using eye-lens AAs, and that is the geographic signal captured in the isotopes can result from either the consumer or its prey. Because some prey types are highly mobile, there is currently no way to distinguish whether geographic influences originate from the consumer or its mobile prey. For example, a high-site-fidelity predator might feed on migratory fishes traveling through its territory (e.g., schooling fishes or squids). Another possibility is that the predator is migratory but its prey has high site fidelity (e.g., benthic invertebrates). Either scenario can cause isotopic variability that originates from geographic differences in baseline δ^15^N. Attributing the source of the baseline variability to mobile prey, mobile consumer, or some combination of both cannot be completed without more information. CSIA-AA of eye lens proteins nevertheless disentangles trophic position-driven differences in δ^15^N from those driven by baseline changes, which is something bulk stable isotope analysis is unable to accomplish.

Considering that the present study is the only the second peer-reviewed study to reconstruct individual CSIA-AA histories and associated lifetime trophic trajectories from fish eye lenses [13], further study of migratory patterns via CSIA-AA is necessary to reveal more detailed trophic and geographic information. It should also be kept in mind that, although isoscapes on the West Florida Shelf present robust gradients, these gradients are somewhat dynamic (i.e., spatial stationarity cannot be assumed) [14], and thus one must be careful not to over-interpret data.

Another limitation is uncertainty about the parameters β and TDF in the TP equation. It has been well established that TDF estimations can vary in response to nutritional and physiological factors. [2,46,47] Different values of β have been observed for marine microalgae, C3 plants, and C4 plants. Although CAM plants do not tend to dominant primary-producer biomass and are unlikely to serve as an aquatic basal resource due to poor hydrologic connectivity between arid and aquatic environments, they do comprise 6% of both the terrestrial and aquatic environments [48]. Therefore it is important to note the value of β for CAM plants and β variation within each of the other primary-producer types have not yet been investigated [4]. Likewise, the accuracy of calculated trophic positions are greatly dependent on the accuracy of the TDF parameter, which has proved to be quite variable [2,6,49,50]. It is important that new techniques are built on a strong foundation, which is why the current study uses glutamic acid and phenylalanine to derive trophic position estimates. Although glutamic acid (trophic AA) and phenylalanine (source AA) are most commonly used for calculating trophic position, Bradley *et al*. [6] demonstrated that the accuracy of trophic position estimates increases when more than one trophic and one source AA are combined in the calculation. Germain *et al*. [46] have also called for the development of not only multi-AA models, but also multi-TDF models as a means of improving the accuracy of trophic position estimates [36]. Currently, the vast majority of TP_CSIA_ calculations assume a simple, additive framework, where the TDF remains constant as trophic position increases (e.g., 3.0 or 3.4‰ increase in bulk δ^15^N per trophic step). However, Hussey *et al*. [36] presented data indicating TDF decreases with increasing trophic position, and that a scaled framework approach based on narrowing TDF with increasing trophic position may more accurately represent organisms at higher trophic positions. They argue that the absolute value of δ^15^N in the food determines TDF, wherein low values inherently fractionate more and high values fractionate less. Hussey et al [36] liken trophic fractionation to food web biomagnification of toxins, the TDF that you observe in a predator depends on the absolute value of its prey.

In summary, we have demonstrated that CSIA-AA can be successfully applied to individual eye-lens laminae to reconstruct lifetime δ^15^N trends. These trends can be used to account for variations in isotopic baselines (via source AAs) and produce more accurate estimates of trophic position throughout life. Furthermore, our application of CSIA on eye lens AAs supports better understanding of long-distance migratory patterns by exploring an additional incremental CSIA-AA record [51,52]. While the current study focuses on marine teleosts, we suggest CSIA-AA of eye lenses can be used to identify isotopic records for other vertebrate taxa, both marine and terrestrial. Although further work is still needed to overcome certain limitations, this general approach has great future potential.

## References

[1] Post DM. Using stable isotopes to estimate trophic position: Models, methods, and assumptions. Ecology. 2002;83(3):703–18. doi: 10.2307/3071875 WOS:000173967500012.

[2] McMahon KW, McCarthy MD. Embracing variability in amino acid delta N-15 fractionation: mechanisms, implications, and applications for trophic ecology. Ecosphere. 2016;7(12):26. ARTN e01511 doi: 10.1002/ecs2.1511 WOS:000390136700015.

[3] Boecklen WJ, Yarnes CT, Cook BA, James AC. On the Use of Stable Isotopes in Trophic Ecology. Annual Review of Ecology, Evolution, and Systematics, Vol 42. 2011;42(1):411–40. doi: 10.1146/annurev-ecolsys-102209-144726 WOS:000299438300019.

[4] Whiteman JP, Smith EAE, Besser AC, Newsome SD. A Guide to Using Compound-Specific Stable Isotope Analysis to Study the Fates of Molecules in Organisms and Ecosystems. Diversity-Basel. 2019;11(1). ARTN 810.3390/d11010008. WOS:000459740700007.

[5] Braun A, Vikari A, Windisch W, Auerswald K. Transamination governs nitrogen isotope heterogeneity of amino acids in rats. J Agric Food Chem. 2014;62(32):8008–13. Epub 2014/07/19. doi: 10.1021/jf502295f . 25036536

[6] Bradley CJ, Wallsgrove NJ, Choy CA, Drazen JC, Hetherington ED, Hoen DK, et al. Trophic position estimates of marine teleosts using amino acid compound specific isotopic analysis. Limnol Oceanogr Meth. 2015;13(9):476–93. doi: 10.1002/lom3.10041 WOS:000361968000003.

[7] Chikaraishi Y, Ogawa NO, Ohkouchi N. Further evaluation of the trophic level estimation based on nitrogen isotopic composition of amino acids. Earth, life, and isotopes. 2010;415:37–51.

[8] Greiling TM, Clark JI. New insights into the mechanism of lens development using zebra fish. Int Rev Cell Mol Biol. 2012;296:1–61. Epub 2012/05/09. doi: 10.1016/B978-0-12-394307-1.00001-1 . 22559937

[9] Dahm R, Schonthaler HB, Soehn AS, Van Marle J, Vrensen G. Development and adult morphology of the eye lens in the zebrafish. Experimental Eye Research. 2007;85(1):74–89. doi: 10.1016/j.exer.2007.02.015 WOS:000248507500010. 17467692

[10] Wallace AA, Hollander DJ, Peebles EB. Stable isotopes in fish eye lenses as potential recorders of trophic and geographic history. PLoS One. 2014;9(10):e108935. Epub 2014/10/04. doi: 10.1371/journal.pone.0108935 ; PubMed Central PMCID: PMC4184832. 25279946 PMC4184832

[11] Peebles EB, Hollander DJ. Combining Isoscapes with Tissue-Specific Isotope Records to Recreate the Geographic Histories of Fish. Scenarios and Responses to Future Deep Oil Spills 2020. p. 203–18.

[12] Granneman JE. Evaluation of Trace-Metal and Isotopic Records as Techniques for Tracking Lifetime Movement Patterns in Fishes [Ph.D.]. Ann Arbor: University of South Florida; 2018.

[13] Harada Y, Ito SI, Ogawa NO, Yoshikawa C, Ishikawa NF, Yoneda M, et al. Compound-Specific Nitrogen Isotope Analysis of Amino Acids in Eye Lenses as a New Tool to Reconstruct the Geographic and Trophic Histories of Fish. Frontiers in Marine Science. 2022;8:9. doi: 10.3389/fmars.2021.796532 WOS:000760853200001.

[14] Radabaugh KR, Hollander DJ, Peebles EB. Seasonal δ13C and δ15N isoscapes of fish populations along a continental shelf trophic gradient. Continental Shelf Research. 2013;68:112–22. doi: 10.1016/j.csr.2013.08.010

[15] Silfer JA, Engel MH, Macko SA, Jumeau EJ. Stable Carbon Isotope Analysis of Amino-Acid Enantiomers by Conventional Isotope Ratio Mass-Spectrometry and Combined Gas-Chromatography Isotope Ratio Mass-Spectrometry. Anal Chem. 1991;63(4):370–4. doi: 10.1021/ac00004a014 WOS:A1991EX23500015.

[16] Ellis GS. Compound-Specific Stable Isotopic Analysis of Protein Amino Acids: Ecological Applications in Modern and Ancient Systems [Ph.D.]. Ann Arbor: University of South Florida; 2012.

[17] Corr LT, Berstan R, Evershed RP. Optimisation of derivatisation procedures for the determination of delta 13C values of amino acids by gas chromatography/combustion/isotope ratio mass spectrometry. Rapid Commun Mass Spectrom. 2007;21(23):3759–71. Epub 2007/11/09. doi: 10.1002/rcm.3252 . 17990247

[18] Metges CC, Daenzer M. 13C Gas Chromatography–Combustion Isotope Ratio Mass Spectrometry Analysis of N-Pivaloyl Amino Acid Esters of Tissue and Plasma Samples. Analytical Biochemistry. 2000;278(2):156–64. 10.1006/abio.1999.4426. doi: 10.1006/abio.1999.4426 10660457

[19] de Busserolles F, Fitzpatrick JL, Paxton JR, Marshall NJ, Collin SP. Eye-size variability in deep-sea lanternfishes (Myctophidae): an ecological and phylogenetic study. PLoS One. 2013;8(3):e58519. Epub 2013/03/09. doi: 10.1371/journal.pone.0058519 ; PubMed Central PMCID: PMC3589346. 23472203 PMC3589346

[20] Lima ARA, Barletta M, Dantas DV, Possato FE, Ramos JAA, Costa MF. Early development and allometric shifts during the ontogeny of a marine catfish (Cathorops spixii-Ariidae). Journal of Applied Ichthyology. 2012;28(2):217–25. doi: 10.1111/j.1439-0426.2011.01903.x WOS:000301051400009.

[21] Quaeck-Davies K, Bendall VA, MacKenzie KM, Hetherington S, Newton J, Trueman CN. Teleost and elasmobranch eye lenses as a target for life-history stable isotope analyses. PeerJ. 2018;6:e4883. Epub 2018/06/12. doi: 10.7717/peerj.4883 ; PubMed Central PMCID: PMC5991300. 29888128 PMC5991300

[22] Herdter ES, Chambers DP, Stallings CD, Murawski SA. Did the Deepwater Horizon oil spill affect growth of Red Snapper in the Gulf of Mexico? Fisheries Research. 2017;191:60–8. doi: 10.1016/j.fishres.2017.03.005

[23] Curtis JS, Albins MA, Peebles EB, Stallings CD. Stable isotope analysis of eye lenses from invasive lionfish yields record of resource use. Marine Ecology Progress Series. 2020;637:181–94. doi: 10.3354/meps13247 WOS:000521739800012.

[24] Simpson SJ, Sims DW, Trueman CN. Ontogenetic trends in resource partitioning and trophic geography of sympatric skates (Rajidae) inferred from stable isotope composition across eye lenses. Marine Ecology Progress Series. 2019;624:103–16. doi: 10.3354/meps13030 WOS:000485737900009.

[25] Kurth BN, Peebles EB, Stallings CD. Atlantic Tarpon (Megalops atlanticus) exhibit upper estuarine habitat dependence followed by foraging system fidelity after ontogenetic habitat shifts. Estuar Coast Shelf S. 2019;225. ARTN 106248 doi: 10.1016/j.ecss.2019.106248 WOS:000478712800025.

[26] Ricci MP, Merritt DA, Freeman KH, Hayes JM. ACQUISITION AND PROCESSING OF DATA FOR ISOTOPE-RATIO-MONITORING MASS-SPECTROMETRY. Organic Geochemistry. 1994;21(6–7):561–71. doi: 10.1016/0146-6380(94)90002-7 WOS:A1994NU29100002. 11539433

[27] Murdoch WW. Switching in General Predators. Experiments on Predator Specificity and Stability of Prey Populations. Ecol Monogr. 1969;39(4):335–&. doi: 10.2307/1942352 WOS:A1969F178500001.

[28] Newsome SD, del Rio CM, Bearhop S, Phillips DL. A niche for isotopic ecology. Frontiers in Ecology and the Environment. 2007;5(8):429–36. doi: 10.1890/060150.1 WOS:000249962100019.

[29] Strelcheck A, Cowan J Jr, Patterson W III. Site fidelity, movement, and growth of red snapper, Lutjanus campechanus: Implications for artificial reef management. Population Ecology and Fisheries of US Gulf of Mexico Red Snapper American Fisheries Society Bethesda, Maryland. 2007:135–48.

[30] Patterson W, editor A review of movement in Gulf of Mexico red snapper: implications for population structure pp. 221–235. Red snapper ecology and fisheries in the US Gulf of Mexico American Fisheries Society, Symposium; 2007.

[31] Harper A, Landing W, Chanton JP. Controls on the Variation of Methylmercury Concentration in Seagrass Bed Consumer Organisms of the Big Bend, Florida, USA. Estuaries and Coasts. 2017;41(5):1486–95. doi: 10.1007/s12237-017-0355-6

[32] Radabaugh KR, Peebles EB. Multiple regression models of delta C-13 and delta N-15 for fish populations in the eastern Gulf of Mexico. Continental Shelf Research. 2014;84:158–68. doi: 10.1016/j.csr.2014.05.002 WOS:000339696200013.

[33] Hyslop EJ. Stomach Contents Analysis—a Review of Methods and Their Application. J Fish Biol. 1980;17(4):411–29. doi: 10.1111/j.1095-8649.1980.tb02775.x WOS:A1980LA21800008.

[34] Dale JJ, Wallsgrove NJ, Popp BN, Holland KN. Nursery habitat use and foraging ecology of the brown stingray Dasyatis lata determined from stomach contents, bulk and amino acid stable isotopes. Marine Ecology Progress Series. 2011;433:221–36. doi: 10.3354/meps09171 WOS:000292890400018.

[35] Seminoff JA, Benson SR, Arthur KE, Eguchi T, Dutton PH, Tapilatu RF, et al. Stable isotope tracking of endangered sea turtles: validation with satellite telemetry and delta 15N analysis of amino acids. PLoS One. 2012;7(5):e37403. Epub 2012/06/06. doi: 10.1371/journal.pone.0037403 ; PubMed Central PMCID: PMC3362573. 22666354 PMC3362573

[36] Hussey NE, Macneil MA, McMeans BC, Olin JA, Dudley SF, Cliff G, et al. Rescaling the trophic structure of marine food webs. Ecol Lett. 2014;17(2):239–50. Epub 2013/12/07. doi: 10.1111/ele.12226 ; PubMed Central PMCID: PMC3912912. 24308860 PMC3912912

[37] Goethel DR, Smith MW. SEDAR 52 Overfishing limits and acceptable biological catches for the red snapper fishery in the US Gulf of Mexico. 2018.

[38] Gallaway BJ, Szedlmayer ST, Gazey WJ. A life history review for red snapper in the Gulf of Mexico with an evaluation of the importance of offshore petroleum platforms and other artificial reefs. Reviews in Fisheries Science. 2009;17(1):48–67.

[39] Cowan J, Grimes C, Patterson W, Walters C, Jones A, Lindberg W, et al. Red snapper management in the Gulf of Mexico: science-or faith-based? Reviews in Fish Biology and Fisheries. 2011;21(2):187–204.

[40] Patterson WF, Wilson CA, Bentley SJ, Cowan JH Henwood T, Allen YC, et al., editors. Delineating juvenile red snapper habitat on the northern Gulf of Mexico continental shelf. American Fisheries Society Symposium; 2005: American Fisheries Society.

[41] McCawley JR, Cowan Jr JH, editors. Seasonal and size specific diet and prey demand of red snapper on Alabama artificial reefs. American Fisheries Society Symposium; 2007.

[42] Szedlmayer ST, Shipp RL. Movement and Growth of Red Snapper, *Lutjanus campechanus*, from an Artificial Reef Area in the Northeastern Gulf-of-Mexico. Bulletin of Marine Science. 1994;55(2–3):887–96. WOS:A1994QB58400050.

[43] Watterson JC, Patterson WF, Shipp RL, Cowan JH. Movement of Red Snapper, *Lutjanus campechanus*, in the North Central Gulf of Mexico: Potential Effects of Hurricanes. Gulf of Mexico Science. 1998;16(1). doi: 10.18785/goms.1601.13

[44] Diamond SL, Campbell MD, Olson D, Wang Y, Zeplin J, Qualia S, editors. Movers and stayers: individual variability in site fidelity and movements of red snapper off Texas. American Fisheries Society Symposium; 2007: American Fisheries Society, 5410 Grosvenor Ln. Ste. 110 Bethesda MD 20814.

[45] Barnes C, Maxwell D, Reuman DC, Jennings S. Global patterns in predator-prey size relationships reveal size dependency of trophic transfer efficiency. Ecology. 2010;91(1):222–32. doi: 10.1890/08-2061.1 WOS:000275458500025. 20380211

[46] Germain LR, Koch PL, Harvey J, McCarthy MD. Nitrogen isotope fractionation in amino acids from harbor seals: implications for compound-specific trophic position calculations. Marine Ecology Progress Series. 2013;482:265–+. doi: 10.3354/meps10257 WOS:000319337100021.

[47] Delibes M, Blazquez MC, Fedriani JM, Granados A, Soriano L, Delgado A. Isotopic Niche Variation in a Higher Trophic Level Ectotherm: Highlighting the Role of Succulent Plants in Desert Food Webs. Plos One. 2015;10(5):17. doi: 10.1371/journal.pone.0126814 WOS:000354545600082. 25973609 PMC4431868

[48] Keeley JE. CAM Photosynthesis in Submerged Aquatic Plants. Botanical Review. 1998;64(2):121–75. doi: 10.1007/BF02856581

[49] Nielsen JM, Popp BN, Winder M. Meta-analysis of amino acid stable nitrogen isotope ratios for estimating trophic position in marine organisms. Oecologia. 2015;178(3):631–42. Epub 2015/04/07. doi: 10.1007/s00442-015-3305-7 . 25843809

[50] Chikaraishi Y, Ogawa NO, Kashiyama Y, Takano Y, Suga H, Tomitani A, et al. Determination of aquatic food-web structure based on compound-specific nitrogen isotopic composition of amino acids. Limnol Oceanogr Meth. 2009;7:740–50. doi: 10.4319/lom.2009.7.740 WOS:000273294600003.

[51] Matsubayashi J, Osada Y, Tadokoro K, Abe Y, Yamaguchi A, Shirai K, et al. Tracking long-distance migration of marine fishes using compound-specific stable isotope analysis of amino acids. Ecol Lett. 2020;23(5):881–90. doi: 10.1111/ele.13496 WOS:000525566900011. 32212213

[52] Magozzi S, Thorrold SR, Houghton L, Bendall VA, Hetherington S, Mucientes G, et al. Compound-Specific Stable Isotope Analysis of Amino Acids in Pelagic Shark Vertebrae Reveals Baseline, Trophic, and Physiological Effects on Bulk Protein Isotope Records. Frontiers in Marine Science. 2021;8:17. doi: 10.3389/fmars.2021.673016 WOS:000696526200001.

