# Correction: Reconstructions of individual fish trophic geographies using isotopic analysis of eye-lens amino acids

**DOI:** 10.1371/journal.pone.0332937

**Published:** 2025-09-19

**Authors:** Amy A. Wallace, Greg S. Ellis, Ernst B. Peebles

The Data Availability statement for this article is incorrect. The correct statement is: Data are publicly available through Digital Commons at https://digitalcommonsdata.usf.edu/ (isotope data – https://doi.org/10.17632/vhmf7w5zyr.1) and the Gulf of Mexico Research Initiative Information & Data Cooperative (GRIIDC) at https://data.gulfreserachinitiative.org (site location – https://doi.org/10.7266/N7X34W1J).
